# Effect of Distorted Visual Feedback on the Sense of Agency

**DOI:** 10.1155/2008/425267

**Published:** 2008-04-11

**Authors:** C. Farrer, M. Bouchereau, M. Jeannerod, N. Franck

**Affiliations:** ^1^Centre de Neuroscience CognitiveCNRSUniversité Claude Bernard Lyon1RSCN69675 LyonFrance; ^2^Centre Hospitalier Le VinatierLyonFrance

**Keywords:** Agency, action awareness, forward model

## Abstract

It has been hypothesized that an internal model is involved in controlling and recognizing one’s own actions (action attribution). This results from a comparison process between the predicted sensory feedback of the action and its real sensory consequences. The aim of the present study is to distinguish the respective importance of two action parameters (time and direction) on such an attribution judgment.

We used a device that allows introduction of discordance between the movements actually performed and the sensory feedback displayed on a computer screen. Participants were asked to judge whether they were viewing (1) their own movements, (2) their own movements modified (spatially or temporally displaced), or (3) those of another agent (i.e, the experimenter). In fact, in all conditions they were only shown their own movements either unaltered or modified by varying amounts in space or time.

Movements were only attributed to another agent when therewas a high spatial discordance between participants’ hand movements and sensory feedback. This study is the first to show that the direction of movements is a cardinal feature in action attribution, whereas temporal properties of movements play a less important role.

## 1. Introduction

Most of our actions are realized in a social context where we have to interact with other people. Appropriate interactions require that each action is continuously and correctly referred to its agent, thus differentiating the actions caused by one self from those caused by others. This ability to refer the origin of an action to oneself (also called "attribution of action" [5,6]) may be based on internal forward models of action. These models have mainly been used to account for aspects of motor control [12], but might also be useful in understanding higher levels of action representation such as the awareness of action and attribution of action [4]. Forward modeling allows the central nervous system to represent the predicted sensory consequences of a movement [11]. This prediction [9] can be compared to the reafferent signals (i.e. sensory feedback signals arising as a consequence of the movement itself). If the perceived sensory changes are correlated with the predicted sensory feedback, they are registered as consequences of one's own action. If not, by contrast, they are registered as originating from an external source [1, 2,4,9]. This comparison process has been considered a key mechanism for referring the origin of an action and thus for attributing an action [4]. However, the influence of the nature or the modality of the information on our ability to attribute an action has never been investigated. Specifically, we wondered whether spatial and temporal information equally influence the capacity to recognize and attribute one's own action.

To answer this question, we used a device that provides the dynamic representation of the movements executed with a joystick, in the guise of a virtual hand holding a joystick [3]. Angular or temporal biases can be introduced in this representation, generating discordance between the movements actually performed and

ISSN 0953-4180/08/$17.00 © 2008 – IOS Press and the authors. All rights reserved the movements displayed on the computer screen. Participants were told that they could see either their own movements 1) unmodified, 2) modified (either deviated or delayed) or 3) the experimenter's movements. In reality, they only saw their own movements with or without a perturbation; the experimenter's movements were never shown to the participants even though they were told the contrary. We hypothesized that for high bias values, participants would experience seeing the experimenter's movements even though in reality they observed their own movements but highly modified. They would therefore misattribute their own movements to the experimenter.

## 2. Material and methods

### 2.1. Participants

Twenty subjects participated in this experiment (mean age 29.8 ± 10.95). They were predominantly right-handed with an average handedness score of 79.05 ± 32.04 according to the Edinburgh questionnaire [7]. All participants gave written informed consent and their rights were protected.

### 2.2. Apparatus

Participants were required to hold a joystick with their right hand and execute random movements. They did not have direct visual input of their own hand but they observed the movements of the joystick via a virtual hand holding a joystick which represented the actual position of the joystick they were physically holding (Fig. 1).

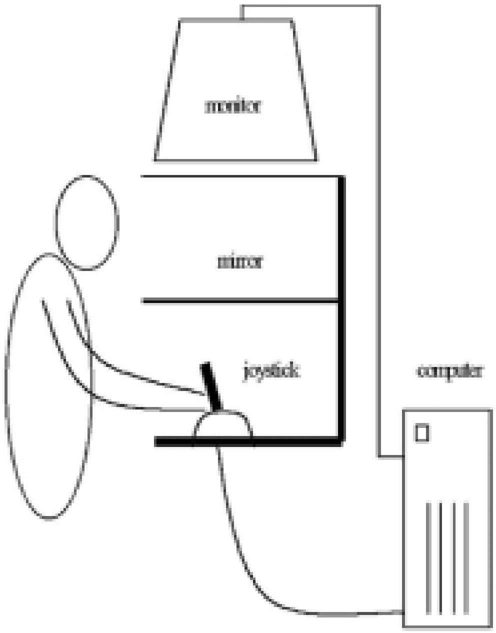

Fig. 1. Schema of the device. The participants sat comfortably in front of the apparatus with their forehead leaning on a foam cushion. A computer screen was placed face down on a metallic support. A horizontal mirror, located below the screen, reflected the image of the screen. A joystick was placed below the mirror on the table supporting the apparatus. The distance between the table and the mirror was 31 cm. The participants had to hold a joystick with their right hand and execute random movements with their elbow resting on the table. The movements of the joystick were fed into a virtual hand holding a joystick according to the real position of the joystick actually held by the participants. The virtual image was projected on a mirror overlying the participants' hand. Thus, when the participants looked at the mirror, they saw the image of a virtual hand moving a joystick just above their own hand actually doing that. This design allowed the dynamic representation of the movements of the joystick held by the participants with an intrinsic delay inferior to 30 ms. The position of the participants' forearm was regained and adjusted so as to coincide with the direction of the virtual forearm seen in the mirror.

### 2.3. Procedure

Each trial started with a dark screen, the image of the virtual hand then appeared for 5 seconds, the participants had to execute random movement with the joystick and direct their attention to the origin of the movement they observed during the entire duration that the image of the virtual hand was present on the screen. Participants then had to answer whether the movements they saw on the screen exactly corresponded with the ones they executed ("self" response), or if they were modified ("bias" response), or if they were not their own but were controlled by the experimenter ("other" response). The verbal responses were recorded by the experimenter. Three categories of trials were used: 1. Neutral trials: movements of the virtual hand exactly replicated those made by the joystick. 2. Trials with angular biases: movements of the virtual hand were deviated either to the right or to the left with respect to those made by the joystick. Thirteen values of angular bias were used (5°, 10°, 15°, 20°, 30°, 40°, 50°, 60°, 70°, 80°, 90°, 100° and 110°). 3. Trials with temporal biases: movements of the virtual hand were delayed by a given time with respect to those made by the joystick. Thirteen values of temporal bias were used (50 ms, 100 ms, 150 ms, 200 ms, 300 ms, 400 ms, 500 ms, 600 ms, 700 ms, 800 ms, 900 ms,1000 ms and 1100 ms). Each value was repeated 10 times resulting in 270 trials. Each participant performed the trials in a different pseudo-random order.

At the beginning of the experiment, participants had a practice session in order to get acquainted with the device. During this practice session they executed random movements and had to say whether the movements they saw corresponded with the ones they executed. This session consisted of three 30-second trials, with or without a bias (0°, 50° or 500 ms), performed in a randomized order.

## 3. Results

### 3.1. Descriptive analysis

In the spatial condition, participants gave the three distinct kinds of responses ("self", "bias" and "other"). These responses were distributed as a function of the bias value: a "self" response was given for the lowest angular biases, a "bias" response for intermediate biases and an "other" response for the highest biases. In the temporal condition, however, participants never gave an "other response". Instead, a "self" response was given for low temporal biases while a "bias" response was given for both intermediate and higher biases (Fig. 2a and b).

### 3.2. Statistical results

For each experimental condition we measured the total number of verbal responses given for each of the three response categories and then performed a repeated measures ANOVA on these data. The factors were: "response" (3 levels: "self", "bias" and "other"), modality (2 levels: angular and temporal) and "value of the bias" (14 levels).

We found a significant main effect of response (F (2, 38) = 86.05, $p < 0.0001$) with participants giving significantly more "bias" responses than "self" and "other" responses, irrespective of the modality of the perturbation. Interaction effects were significant between the response and the value of the bias (F(26, 494) = 165.11, $p < 0.0001$), the response and the modality (F(2, 38) = 188.40, $p < 0.0001$) and between these three factors (F(26, 494) = 64.63, $p < 0.0001$). These results indicate that the response given by the participants depends not only on the value of the bias but also on the modality of the perturbation. Post-hoc analyses with Scheffé's tests revealed that the participants gave significantly more "bias" responses in the temporal modality than in the angular modality ($p < 0.0001$) whereas they gave more "other" responses in the angular modality than in the temporal modality ($p < 0.0001$). However no significant differences were found for the "self" responses between these two modalities.

We also determined the bias values at which the kind of response given by the participants significantly changed. For each response category of each condition, t-tests for pairwise comparisons were performed between the different bias values. In the spatial condition, participants gave more "self" responses for biases between 0° and 15°, more "bias" responses for biases between 20° and 30° ($p < 0.05$) and more "other" responses for biases between 50° and 60° ($p < 0.05$) (Fig. 2a). These results indicate that a first transition in the kind of response given by the participants ("self" vs. "bias" response) occurred for bias values between 15° and 20° and a second transition ("bias" vs. "other" responses) occurred for bias values between 30° and 50°.

In the temporal condition, participants gave significantly more "self" responses for biases between 0 ms and 50 ms ($p < 0.05$) and more "bias" responses for biases between 150 ms and 1100 ms ($p < 0.05$) (Fig. 2b).

## 4. Discussion

This study aimed at evaluating the parameters involved in action attribution. Consistent with previous reports of the influence of a mismatch between intention and perceived consequences on action attribution, with participants no longer experiencing themselves as the author of the action [8,10], we showed that an important modification of one's own action led participants to attribute this action to another person. The present results, however, further reveal that the modality of the perturbation also influences the judgment of attribution. Specifically, modifying the apparent direction of the movement actually performed, by introducing an important spatial deviation (⩾50°), results in the attribution of one's own actions to another person. However, even with extremely long delays (1100 ms), actions were almost never misattributed. The directional information seems thus more important for attributing an action to oneself or to another person than the timing cues.

How can this difference between spatial and temporal discordance be explained? The recognition of an action as one's own necessitates matching the final goal of the action with our intended action. We propose that the difference obtained between angular and temporal

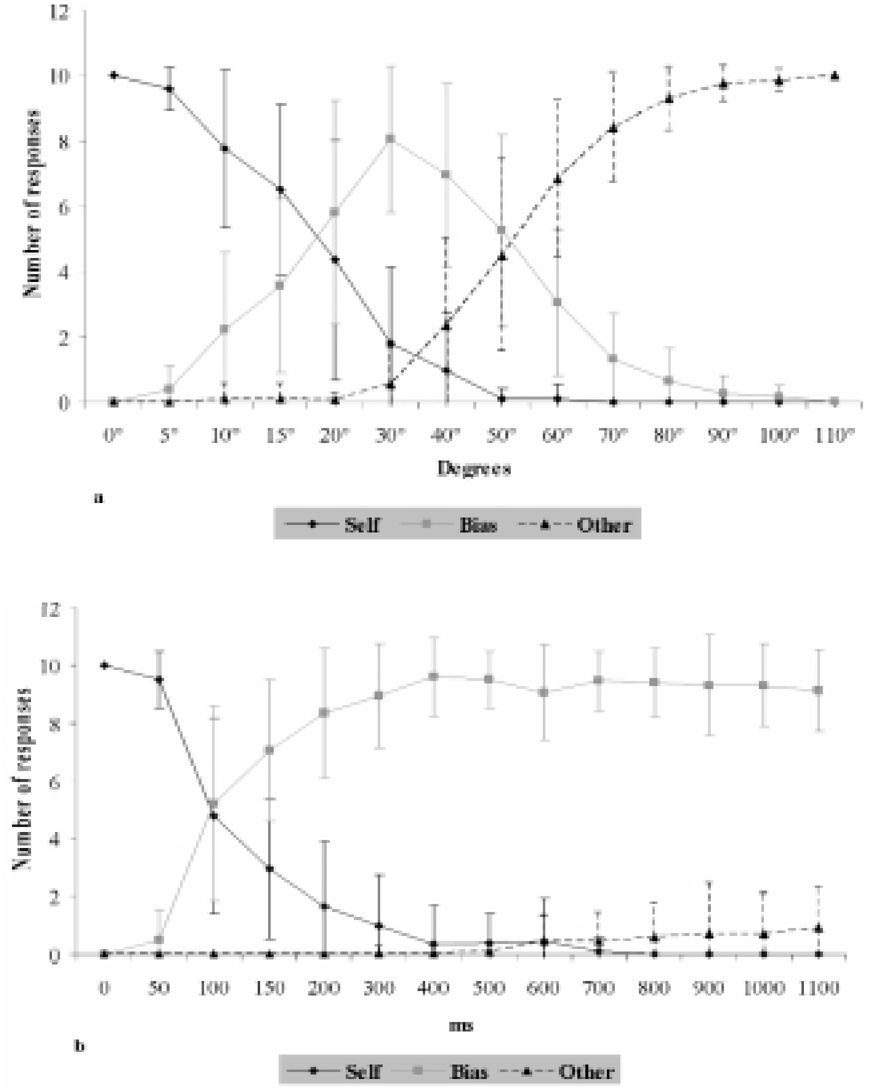

Fig. 2. (a) Means and standard deviations of the number of "self" responses, "bias" responses and "other" responses as a function of the angular bias. (b) Means and standard deviations of the number of "self" responses, "bias" responses and "other" responses as a function of the temporal bias.

modality on a misattribution of one's own actions to another agent may be explained by a differential influence of spatial and temporal information on the perception of the goal of a movement. When the visual feedback of the action is perturbed, there is a mismatch between these two kinds of information. For a large enough mismatch, participants became aware of the deviation or of the delay but they misattributed their own actions only for high spatial deviations. A modification of the apparent direction of the movement performed leads to the perception of a goal that differs from the one prior to the execution. An important delay between the movement executed and its visualization does not result in the perception of different goals. An important deviation would thus lead one to attribute one's own movements to another person because the perception of the final goal would no longer correspond to the goal of the intended action.

## Acknowledgments

The authors thank Scott Johnson-Frey and Karen Reilly for their fruitful comments on the manuscript. This study was supported by the Medical Research Foundation (FRM).
